# Can we enhance the ability to return to work among workers with stress-related disorders?

**DOI:** 10.1186/1471-2458-9-372

**Published:** 2009-10-05

**Authors:** Flemming Lander, Claus Friche, Helle Tornemand, Johan Hviid Andersen, Lilli Kirkeskov

**Affiliations:** 1 Dept of Occupational Medicine, Regional Hospital Herning, Herning, Denmark; 2 Clinic of Occupational and Environmental Medicine, Bispebjerg University Hospital, Copenhagen NV, Denmark

## Abstract

**Background:**

Stress-related disorders are widespread and responsible for high societal costs e.g. sick leave payment and reduced productivity. The aim of this study was to evaluate the effect of an intervention program on return to work or labour market.

**Methods:**

In a controlled interventional study design we compared 72 emotionally distressed patients, who received support during 2006, with 89 control individuals who had also been sick listed for emotional distress. Intervention was provided by trained psychologists and social workers who were in continuous dialog with the patients, providing counselling e.g. on decisions concerning resumption of work, support to families, participation in meetings with the workplace. Basically, the controls and the intervention group share the same access to welfare benefits. The main outcome was time to return to labour market (TTR).

**Results:**

The baseline characteristics were similar in the two groups. There were no differences in the rate of resuming work between the two groups. About 80% in both groups had returned to the labour market after one year.

**Conclusion:**

An intervention program with psychological stress management and case management did not improve work capability compared to usual care. Work resumption as a single outcome probably is an insensitive parameter of intervention management quality, and should be supplemented by other data on different aspects of treatment.

## Background

In western countries the number of persons on sick leave, as well as sick leave expenses due to mental health problems, has rapidly increased in the last two decades and is considered a major source of societal costs [1,2]. Subsequently, during recent years, there has been an increased focus on programs for preventing or reducing work related mental distress in order to improve the psychosocial work environment and lower the derived costs [1,3]. Several types of intervention programs have been studied and a small overall positive effect on individual complaints, psychological resources and responses, and perceived quality of work life has been found [1]. But, in respect to work resumption, study results are far less consistent. This is probably because it has remained unclear how individuals with mental distress disorders should best be treated in order to improve functioning and because of national differences in labour market regulations and official sick leave policy [2,4-6].

In Denmark it is generally held that early supportive efforts that are optimally coordinated between stakeholders, which is the patient, her/his workplace, municipal case worker, and the general practitioner (GP), is the main tool for preventing long-term absenteeism. Thus, poorly coordinated treatment and support is believed to be the main reason for delayed return to work.

We are well aware that the very term 'stress' is ambiguous since it is often used to describe both cause and consequences. There is no scientific agreement on the conceptual basis of 'stress', neither of its definition, assessment, or its potential relationship with work [6]. These problems are not addressed in this study, where we have collapsed all minor mental health problems to be synonymous with stress-related disorders in an otherwise healthy and labour active setting. The aim of this study was to evaluate the effect of an intervention program compared to usual welfare benefit care on return to work or labour market.

## Methods

### The national sick leave policy and primary health care system

The official Danish sick leave policy is based on the obligation of the municipalities to assess all cases of sickness benefits within eight weeks after the first day of sick leave. Thereafter they must make follow-up assessments every fourth week in complicated cases and every eight weeks in uncomplicated cases. At follow-up, the municipal social worker must verify that the sick-listed individual is entitled to receive benefit, i.e. suffers from a medical condition, and, if necessary, establish activities to improve or retain the sick-listed worker's labour market attachment. The assessment must be based on updated medical, social, and vocational information, and it should take place in cooperation with the sick-listed worker, the employer, medical experts, vocational rehabilitation institutions, and other relevant agents. To promote a swift return to work, the municipal social worker can initiate various vocational rehabilitation measures and vocational services. These measures include reduced working hours with supplementary sickness benefits, financial support for workplace adaptations and aids, testing of work ability, job counseling, wage-subsidized job training, and educational measures [7]. Unfortunately though, stakeholders do not always follow procedures or adhere to plans.

Employers' responsibility for sick-listed workers is relatively limited. Thus, employers are only responsible for the financing of sickness benefits for the first two weeks of a sick leave period, while sick leave exceeding two weeks and disability benefits are financed by public authorities [7]. Workers can normally receive sickness benefits for up to 12 months within a period of 18 months.

Furthermore, "care as usual" in Denmark includes free and unlimite access to family based GP's and there is no other alternative primary health care systems e.g. a parallel company based health care system. The GP is the key figure for patients and he/she is responsible for health care initiatives e.g. referral to hospital treatment or other specialized treatments. Thus, in general the GPs are often involved early during their patient's sick leave absence, but they are not legally required to be involved. In some instance public or workplace subsidised help from psychologists is an additional possibility too.

### Sick leave intervention

In 2005, the Department of Occupational Medicine (DOM), Regional Hospital Viborg launched a project aimed at strengthening the co-ordination between stakeholders, combined with a number of consultations with a psychologist and a social worker from the Department, who also were responsible for providing feedback to GPs, workplaces and municipal authorities. The project received funding from the Ministry of Labour and the Municipality of Viborg for an initial period of 2 years.

The intervention involved individual consultations with one of 5 trained psychologists attached to the project and was similar to the methods used by van der Klink [2,3]. Sick leave due to minor mental health problems is considered not only to be a sign of failure to cope, but may also involve elements of avoidance coping. Persistent avoidance is thought to be the main reason for prolonged sickness, thus psychological treatment was primarily directed towards activating and supporting the patients efforts to adopt a problem-solving approach to their problems.

Parallel to psychoeducative treatment, the social worker at the Department provided advise and support to patients e.g. concerning legal matters, and not least various ways of resuming work, e.g. with reduced work hours in an initial period. The social workers also provided support to families, facilitated contacts with work places and participated in meetings with employers.

### Study design and populations

**Figure 1:**
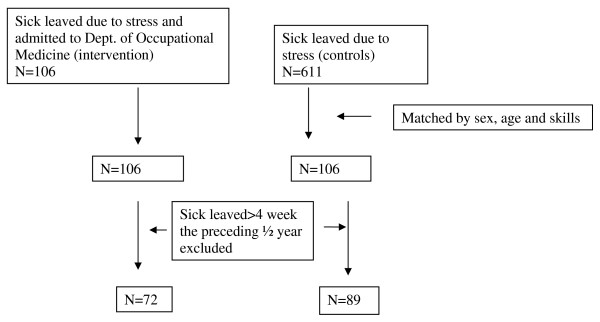
**Flowchart of the intervention group and the controls sick leaved 2006 due to emotional distress**.

A controlled follow up trial was performed among two regionally separated groups on sick leave due to mental distress during 2006 (figure 1). Between January and December 2006 106 individuals consecutively referred to the DOM because of emotional distress and on sick leave were included in the study. The majority was referred to the Department through their GP, but a few were referred from workplaces or labour unions. Before and during the project period all regional GPs were repeatedly informed about the stress management offer for their patients together with the recruitment criterias which were fixed. These consisted of self reported stress without severe mental disorders, no drug or alcohol abuse, and being job active. Further, the importance of immediate intervention management was announced to all referral stakeholders too, especially the regional GPs.

The control group consisted of individuals living in the neighbouring municipality (Randers), which resembles the municipality of Viborg, and who had their first sick leave day in the year 2006. Diagnostic data was obtained from the sick leave files that case workers routinely compiled on the basis of the sick person's self reported reason for their sickness absence. This administrative routine was different compared to other municipalities, but in turn provided a unique opportunity to gather a control group who had received "care as usual". We know that no specific and comprehensive "back to work" intervention program had taken place in the municipality during the observation period. The sick leave files of Randers municipality contained 611 individuals, who were on sick leave due to emotional distress in 2006. Both intervention and control individuals shared the same conditions in respect to legal case work and follow-up by the municipal authorities.

The individual records from the intervention and the control group, a total of 717 individuals, were combined and subsequently merged with the DREAM database which contains information on all social benefit payments for all Danish citizens since 1991 on a weekly basis [8]. Additionally, the register contained information on gender, civil status, ethnic background and nationality, and labour union membership. Records were linked on the basis of the Central Population Register number, which is a 10-digit unique identifier of all Danish citizens. In the next step, each of the 106 individuals in the intervention group was matched to a control individual on gender, age and labour union membership. The match was done manually, but randomly. In general, individuals who share the same labour union membership have both similar type of work and level of vocationally orientated education. All 17 labour unions involved were transformed and dichotomized into unskilled and skilled work (blue collar workers) or into work which demands middle or high level education (white collar workers). In the final step we excluded individuals in both study groups with preceding long term sick leave defined as absence from work of more than 4 weeks during the past half a year before index day. The final study population consisted of 72 intervention individuals and 89 controls.

### Outcomes

Time to return to labour market (TTR) for the intervention group was based on the day where the Department of Occupational Medicine received the referral and the first day of sick leave was used for the control group (index day). The outcome measure was the number of weeks from index day to full return to work or transfer from public health-related benefits to labour-market-related benefits. For survival statistics Kaplan-Meier and Cox regression were used. Analysis was performed using STATA software.

### Ethic

The project was not a randomized controlled clinical trial and thus approval from the ethic committee was not claimed cf. "Guidelines about Notification etc. of a Biomedical Research Project to the Committee System on Biomedical Research Ethics" from The Danish National Committee on Biomedical Research Ethics [9]

## Results

The two groups were quite similar with regards to age, sex, education, marital status and nationality (table 1). The number of consultations with the psychologist and social worker were on average between 4-5 times. The median duration of treatment lasted a little more than half a year. The number of consultations and duration of treatment had a wide range, reflecting different levels of distress and different intervention requirements (table 1).

**Table 1:** Characteristics of the study sample

	**Intervention group N = 72**	**Controls N = 89**
*Demographic variables*		
Age [mean (S.D.)]	42.9 (8.6)	43.1 (8.4)
Sex (% female)	80.6	83.2
Eduvational level (%)		
Unskilled or skilled workers	58.3	52.8
Middle or high educated workers	41.7	47.2
With partnership (%)	59.7	66.3
Danish nationality (%)	97.2	94.4
*Intervention variables*		
Duration of treatment (mean, range days)	156 (4-347)	-
Number of consultations (mean, range)	5.3 (1-11)	-

At the group level both groups shared the same and stable level of sick leave, around 3-7%, the preceeding 3 years before the index day.

**Figure 2:**
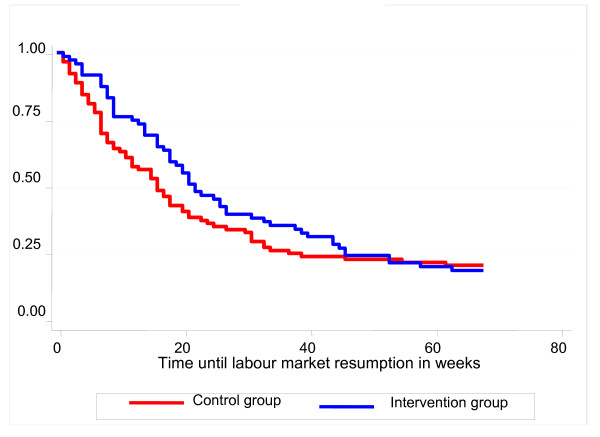
**Probability of not resuming labour market within 68 weeks after the index day**.

Figure 2 shows the probability of not resuming labour market activity within 68 weeks from index day in the intervention and control group. No difference was observed between groups. Cox regression analysis yielded a hazard ratio (HR) of 0.84 (95% CI: 0.60 to 1.19) for TTR.

## Discussion

Among participants who suffer from emotional distress, psychological disease management intervention did not seem to improve TTR within the first year compared to usual care. In this study the psychoeducative management intervention was combined with comprehensive coordinative efforts among stakeholders at the workplace, municipal case workers, and GPs. There are several possible reasons for not finding an intervention effect. First, the prognosis of return to work due to mild mental disorders did not differ between groups. As in similar Dutch studies, about 75-80% of the present study sample had returned to the labour market within one year [4]. Both the Dutch studies and the present study included a mixed job population with mild mental disorders. In many ways the Netherlands is very similar to Denmark concerning well-fare programs and labour market policies. This indicates that in comparable developed countries spontaneous recovery from mild emotional distress or minor mental disorders within a reasonable time fails for 20%. These are probably at risk for having a more serious prognosis. Second, the highly specialized intervention strategy was no more effective than usual care, which could indicate that usual care represents a well functioning welfare system. Third, patients are afraid of losing their job due to prolonged sick leave and return before full recovery. In Denmark, labour market agreements are based on the model of flexicurity, this means flexibility and security at the same time. Flexicurity is an attempt to unite these two fundamental needs in promoting a combination of a flexible labour market and individual income security in order to maintain the Scandinavian social welfare model. In the current context, this means that employers can easily and with short notice dismiss employees without any explanation and without financial compensation [7]. The income security consists of unemployment compensation for at least 4 years, or other temporary benefit payments in a relatively long span of time. But for most persons who are active in the labour market, sick leave or other benefits imply markedly reduced earnings and an elevated risk of prolonged unemployment. Thus, absence from work, even for a short time period, might have serious implication for the patient and her/his family, and thus, she/he will try to avoid this risk and return to work as soon as possible, even if not fully recovered. A comparative study of private sector workers sick-listed for at least 3 months found that 50 percent of Danish workers were dismissed compared to only 11 percent of Dutch workers [7]. In the Netherlands, employers have a much greater responsibility for the reintegration process compared to Danish employers. It was especially easy for Danish employers to dismiss white-collar workers: if specified in the employment contract, employers could dismiss a worker with one-month's notice when the worker had been sick-listed for 120 days within one year [7].

In sum, TTR as a single outcome seems to be an insensitive parameter of intervention management quality. Work ability or capacity is a complex matter and an individual decision of work resumption is not only determined by their mental health status or level of well being but by the impact of specific societal relationships too e.g. current risk of being dismissed, access to suitable sick leave payments or market conditions in general. Our finding is in accordance with a recent review of mental health and work which found limited and conflicting evidence that stress management interventions improve sickness absence rates or return to work [6]. But even though the intervention did not seem to have an effect on TTR, this does not imply that intervention is useless. The intervention group may well have improved their coping skills for future problems which might appear in the period after they had returned to work, e.g. in getting sick due to stress to a lesser extent than those who only expierenced usual welfare benefit care. Thus, future intervention studies within this area should focus on long term work stability, achieved coping skills, well-being and psychological improvements as well as TTR. Besides possible benefit to individual persons the intervention might have provided more lasting improvements in coordination between stakeholders at workplaces, municipal case worker *s*, and GP's. In a variety of western countries, GP's workload related to patients with minor mental distress has increased and they have reported insufficient time for treatment and appropriated support [4]. The present study certainly relieved the GPs by providing the possibility of referral to psychologists and social workers at the regional DOM.

We are well aware that the perfect interventional study trial should include randomization of participants, but this was not possible in our study setting. On the other hand, our study design is free of any ethical aspects and some sort of selection bias which are of great concern when trying to establish randomized study groups where one group receives good care and the controls gets nothing despite an obvious need for support. In reality a randomized study design focused on distress support is very hard to establish and very few researchers have succeeded in carrying them out [2,4,10]. A strength of this study is the very reliable register information on weekly benefit payments compared to other sources of information e.g. self report [8].

A main limitation of the study is that we have no baseline information of risk factors at work and privat life as well as information of mental health disorders on the control group besides self reported stress obtained by the municipal authorities. In general, patients referred to hospitals by their GP might be considered more sick than the participants from our control group. Thus, this difference in the recruitment strategy of our study might imply an underestimation of intervention effects. But if selection bias in health is a severe problem this ought to be reflected in a higher sick leave absence in the years prior to onset of distress in the intervention group compared to the control group [11]. We found no evidence of this. The two groups displayed a rather steady and similar pattern of baseline absence from work and the weekly sick leave prevalence during the preceding 3 years for both study groups was around 5%, which is comparable to the national average [8]. Furthermore, this strong and stable connection to the labour market by the study populations indicates that nobody in the study groups suffered from major illness e.g. severe mental illness, although minor differences between groups can not be excluded.

## Conclusion

This intervention program with psychoeducation and case management of individuals suffering from emotional distress did not seem to be better at preventing long-term absenteeism compared to usual welfare benefit care. However, work ability or capacity is a complex matter and individual decisions about work resumption are not only determined by mental health improvement or level of well being, but also by the impact of specific societal relationships too.

## Competing interests

The authors declare that they have no competing interests.

## Authors' contributions

FL prepared the study plan, contributed to analysis plan, conducted the analyses, wrote the first version and prepared the final version of the manuscript. CF, HT, JHA, and LK prepared the study plan, made the application for funding, contributed to the intervention and to the manuscript. All authors read and approved the final manuscript.

## Pre-publication history

The pre-publication history for this paper can be accessed here:


